# Serological Analysis Identifies Consequential B Cell Epitopes on the Flexible Linker and C-Terminus of Decorin Binding Protein A (DbpA) from Borrelia burgdorferi

**DOI:** 10.1128/msphere.00252-22

**Published:** 2022-07-25

**Authors:** Elaheh Movahed, David J. Vance, Dylan Ehrbar, Greta Van Slyke, Jennifer Yates, Karen Kullas, Michael Rudolph, Nicholas J. Mantis

**Affiliations:** a Division of Infectious Disease, Wadsworth Center, New York State Department of Health, Albany, New York, USA; b New York Structural Biology Center, New York, New York, USA; University of Florida

**Keywords:** Lyme disease, antibody, epitope, vaccine, Borrelia burgdorferi, antibody function

## Abstract

Decorin binding protein A (DbpA) is a surface adhesin of Borrelia burgdorferi, the causative agent of Lyme disease. While DbpA is one of the most immunogenic of B. burgdorferi’s nearly 100 lipoproteins, the B cell epitopes on DbpA recognized by humans following B. burgdorferi infection have not been fully elucidated. In this report we profiled ~270 B. burgdorferi-seropositive human serum samples for IgM and IgG reactivity with a tiled DbpA 18-mer peptide array derived from B. burgdorferi *sensu stricto* strains B31 and 297. Using enzyme-linked immunosorbent assays (ELISA) and multiplex immunoassays (MIA), we identified 12 DbpA-derived peptides whose antibody reactivities were significantly elevated (generally <10-fold) in B. burgdorferi-seropositive sera, compared to those measured in a healthy cohort. The most reactive peptide (>80-fold IgG, 10-fold IgM) corresponded to residues 64 to 81, which map to an exposed flexible loop between DbpA’s α-helix 1 and α-helix 2. This loop, whose sequence is identical between strains B31 and 297, overhangs DbpA’s substrate binding pocket. A second strongly reactive antibody target (>80-fold IgG, 3 to 5-fold IgM) mapped to DbpA’s C-terminus, a lysine rich tail implicated in attachment to glycosaminoglycans. We postulate that antibody responses against these two targets on DbpA could limit B.burgdorferi’s ability to attach to and colonize distal tissues during the early stages of infection.

**IMPORTANCE** The bacterium, Borrelia burgdorferi, is the causative agent of Lyme disease, the most reported tick-borne illness in the United States. In humans, clinical manifestations of Lyme disease are complex and can persist for months, even in the face of a robust antibody response directed against numerous B. burgdorferi surface proteins, including decorin binding protein A (DbpA), which is involved in the early stages of infection. In this study we employed ~270 serum samples from B. burgdorferi-seropositive individuals to better understand human antibody reactivity to specific regions (called epitopes) of DbpA and how such antibodies may function in limiting B. burgdorferi dissemination and tissue colonization.

## INTRODUCTION

The bacterium Borrelia burgdorferi *sensu lato* (B. burgdorferi s.l.) is the causative agent of Lyme disease (LD) in the Northern Hemisphere, with Borrelia burgdorferi *sensu stricto* (B. burgdorferi s.s.) being the most reported tick-borne illness in the United States. In the absence of antibiotic intervention, LD can progress from a localized infection in the first days and weeks following a tick bite to disseminated manifestations (e.g., neuroborreliosis, carditis) and/or Lyme arthritis months or even years later (1). B. burgdorferi infection is accompanied by a robust, antigen-specific serum IgM and IgG response that arises within days. In fact, LD diagnostics involve tiered IgM and IgG serologic assays to measure reactivity against a combination of B. burgdorferi sonicate, B. burgdorferi proteins, and/or peptides (2–4). From the standpoint of immunity, B. burgdorferi-specific serum antibodies are critical both in clearing B. burgdorferi through complement-dependent and complement-independent borreliacidal activities (5–7) and in Fc-mediated opsonophagocytosis (8, 9). However, the specific antibody subsets that contribute to bacterial clearance and the resolution of LD remain unknown (6).

Decorin binding protein A (DbpA; BBA24) is a highly immunoreactive B. burgdorferi protein, as evidenced by the appearance of high titer anti-DbpA serum IgG antibodies in the early stages of experimentally infected mice (10, 11), nonhuman primates (12), and human Lyme disease patients (13). Indeed, anti-DbpA IgM and IgG responses have diagnostic value in LD (14). DbpA is a helical, surface-displayed lipoprotein of ~19 kDa that promotes B. burgdorferi attachment to connective tissues and components of the extracellular matrix (ECM), including glycosaminoglycans (GAGs), such as decorin, dermatan sulfate, and heparin (15–24). By virtue of its ability to adhere to GAGs, DbpA influences B. burgdorferi tropism for specific tissues and cell types (22, 25). DbpA is expressed early during infection and stimulates the onset of antibodies in the absence of CD4 T cell help (26). In a mouse model, anti-DbpA antibodies confer protection against a B. burgdorferi challenge by needle injection, although there is some debate as to whether the same holds true in a natural (tick) route of infection (10, 11, 18, 27). Thus, the role of anti-DbpA antibodies in limiting B. burgdorferi dissemination and colonization remains unresolved.

Despite DbpA being a primary target of the humoral immune response following B. burgdorferi, little is known about the specific epitopes on DbpA recognized by human patients. Arnaboldi and colleagues identified a 15-mer peptide corresponding to N-terminal residues (~6 to 30) of DbpA that was reactive with serum IgM (but not IgG) from early LD patients (28). It should be underscored that those antibody profiles were derived from individuals who had been clinically diagnosed as having erythema migrans (EM), a hallmark of early-stage Lyme disease. Another study identified a DbpA-derived peptide (residues 57–71) reactive with serum IgG antibodies from Lyme neuroborreliosis patients, although the sample size in that study was rather limited (29). Considering DbpA’s overall immunogenicity in humans and the fact that B cell epitope prediction tools, such as Bepipred, identify several DbpA peptides with a high propensity to be antibody targets (30, 31), we sought to revisit the question of linear B cell epitopes on DbpA. Addressing this question was possible because we had access to a large collection of de-identified serum samples that had been designated seropositive for B. burgdorferi antigens via approved diagnostic tests. While the diagnostic tests are not necessarily indicative of Lyme disease, they do afford a high degree of confidence that an individual had experienced a B. burgdorferi infection.

Here, we report the screening of ~270 B. burgdorferi-seropositive serum samples against a tiled DbpA 18-mer peptide array derived from B. burgdorferi strains B31 and 297. One of the most reactive peptides in our collection (A7) corresponds to the conserved flexible linker that overhangs DbpA’s lysine-rich ligand binding pocket. Equally reactive were peptides corresponding to the C-terminal tails of DbpA from B31 and 297, which have also been implicated in substrate recognition. The presence of antibodies targeting these regions of DbpA would be expected to block DbpA-mediated substrate recognition and limit B. burgdorferi colonization of distal tissues.

## RESULTS

### IgM and IgG reactivity with DbpA in B. burgdorferi-seropositive serum samples.

**FIG 1:**
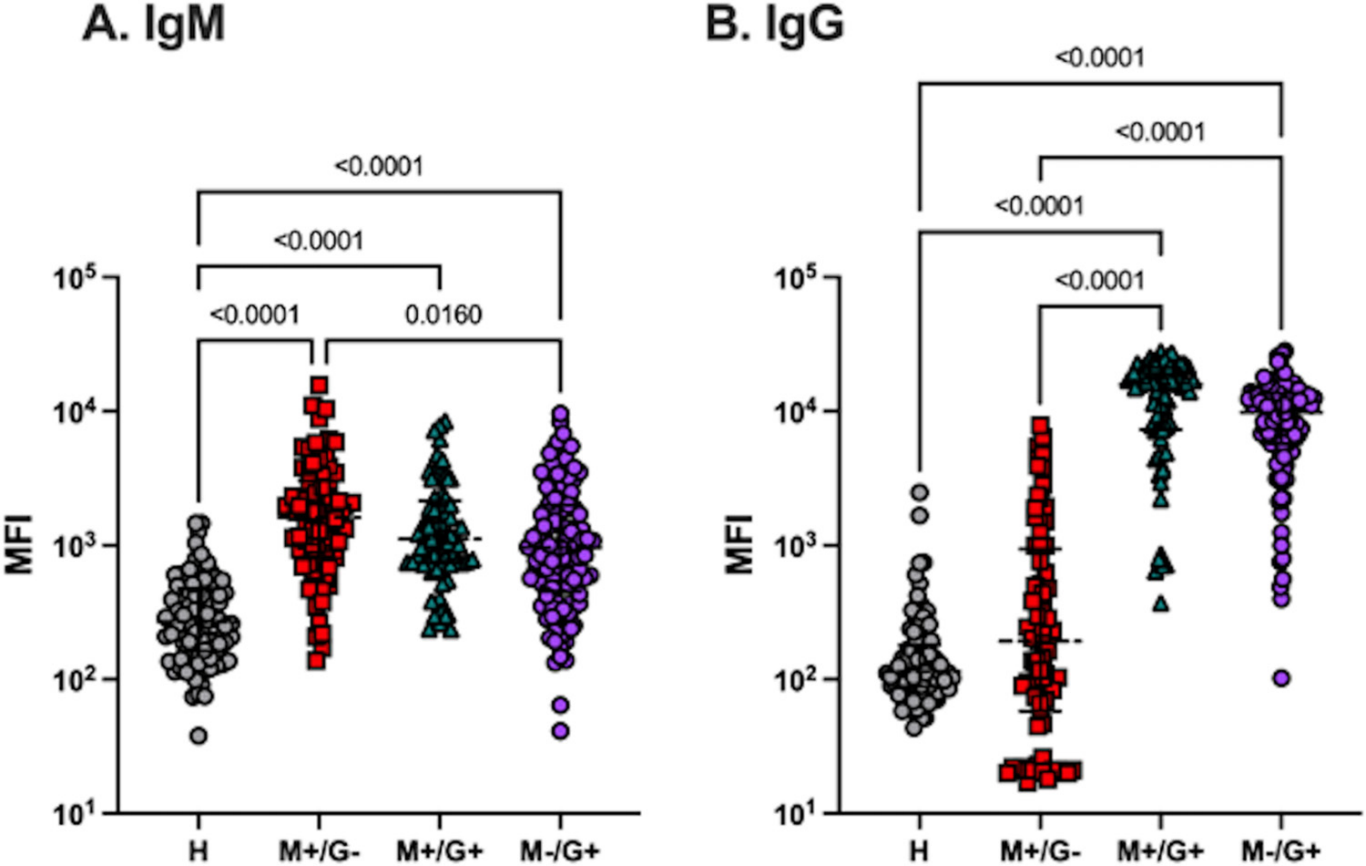
Serum IgM and IgG reactivity with DbpA. Anti-DbpA (A) IgM and (B) IgG reactivity (MFI) in healthy (*n* = 86) or B. burgdorferi-seropositive serum samples, as defined as IgM^+^/IgG^−^ (*n* = 78), IgM^+^/IgG^+^ (*n* = 71), or IgM^−^/IgG^+^ (*n* = 114). Significance was determined by Kruskal-Wallis test with Dunn’s post hoc test.

DbpA is one of the most immunogenic B. burgdorferi proteins in humans and nonhuman primates (12, 13, 32–34). To assess the relative reactivity of DbpA in our collection of ~270 clinical samples, serum samples classified as IgM^+^/IgG^−^, IgM^+^/IgG^+^, and IgM^−^/IgG^+^ reactive were subjected to a Luminex analysis with DbpA conjugated microspheres (Fig. 1). Reactivity was compared to a commercial panel of 87 serum samples obtained from healthy individuals.

Within our panel of B. burgdorferi-seropositive serum samples, anti-DbpA IgM levels (MFI) in all three clinical cohorts (IgM^+^/IgG^−^, IgM^+^/IgG^+^, and IgM^−^/IgG^+^) were significantly elevated (4 to 7-fold) over those of the healthy controls, with the highest MFIs found in the IgM^+^/IgG^−^ group (Fig. 1; Table S1). In the case of IgG, anti-DbpA levels (MFI) were not significantly above those of healthy controls in the IgM^+^/IgG^−^ cohort, but they were markedly elevated in the IgM^+^/IgG^+^ (~70-fold) and IgM^−^/IgG^+^ (~50-fold) cohorts (Table 1; Fig. 1). This profile is consistent with the development of DbpA IgM and IgG antibodies that begins in the early stages of B. burgdorferi infection. Moreover, on an individual sample basis, anti-DbpA IgG levels were greater than IgM levels in the IgM^+^/IgG^+^ and IgM^−^/IgG^+^ cohorts, which is indicative of the maturation of the immune response with time (Fig. S1).

**TABLE 1:** DbpA peptide reactivity with IgG from Lyme disease patient serum samples

		Healthy	IgM+/IgG^−^			IgM+/IgG+			IgM^−^/IgG+		
AA^*a*^	#^*b*^	MFI (SD)	Index (SD)	MFI (SD)	*P* value	Index (SD)	MFI (SD)	*P* value	Index (SD)	MFI (SD)	*P* value
26-188		205.3 (324.9)	4.2 (7.7)	871.5 (1575)	0.85	68.8 (37.7)^*c*^	14128 (7750)	**<0.0001**	48.2 (28.94)^*c*^	9887 (5940)	**<0.0001**
28-45	B10	94.88 (85.49)	1.6 (3.1)	159.1 (297.2)	>0.99	4.8 (6.8)	452.5 (650.4)	**<0.0001**	3.4 (5.9)	323.5 (563.5)	**<0.0001**
37-54	B11	74.50 (59.93)	4.6 (6.4)	347.0 (481.4)	**<0.0001**	9.1 (12.5)	677.9 (934.0)	**<0.0001**	9.4 (10.86)	704.1 (809.1)	**<0.0001**
46-63	A5	216.0 (467.9)	3.1 (3.8)	686.7 (811.7)	**<0.0001**	8.3 (7.9)	1797 (1717)	**<0.0001**	7.7 (10.34)	1659 (2234)	**<0.0001**
55-72	A6	128.8 (61.46)	0.4 (0.6)	55.36 (79.79)	**<0.0001**	3.4 (7.0)	441.6 (902.5)	0.1801	2.4 (5.3)	304.8 (693.8)	0.2298
	C1	168.9 (115.8)	5.6 (15.8)	944.3 (2675)	**<0.0001**	6.9 (8.5)	1176 (1443)	**<0.0001**	5.7 (8.3)	967.8 (1414)	**<0.0001**
64-81	A7	33.72 (15.07)	2.5 (4.1)	85.67 (137.1)	0.47	84.6 (126.1)^*c*^	2853 (4251)	**<0.0001**	59.7 (86.22)^*c*^	2013 (2907)	**<0.0001**
118-135	B1	174.8 (111.6)	3.7 (4.4)	655.2 (775.8)	**<0.0001**	10.0 (6.5)	1754 (1143)	**<0.0001**	9.5 (8.8)	1658 (1543)	**<0.0001**
136-153	B3	164.6 (352.5)	3.9 (5.9)	644.5 (987.0)	**<0.0001**	7.4 (5.1)	1225 (842.5)	**<0.0001**	7.3 (7.7)	1201 (1276)	**<0.0001**
	C4	71.44 (138.9)	7.2 (9.7)	514.4 (697.1)	**<0.0001**	12.9 (9.4)	924.3 (678.4)	**<0.0001**	15.7 (16.81)	1120 (1201)	**<0.0001**
163-180	C6	29.44 (13.68)	1.3 (2.9)	37.65 (87.01)	0.0098	4.3 (11.8)	127.3 (347.0)	**<0.0001**	3.7 (8.7)	109.8 (257.8)	0.1739
172-189	B7	408.5 (187.7)	0.4 (1.3)	198.3 (551.8)	**<0.0001**	2.9 (5.7)	1213 (2348)	>0.9999	2.9 (5.6)	1194 (2314)	0.0317
	C7	44.28 (30.71)	2.5 (4.2)	108.7 (187.5)	0.79	83.5 (122.1)^*c*^	3698 (5407)	**<0.0001**	58.3 (86.03)^*c*^	2583 (3810)	**<0.0001**

a Amino acid residues. Residues 26-188 (top row) refers to full length recombinant DbpA used in this study that lacks the first 25 residues.

b Peptide names, as noted in Fig. 2. Underlines indicate 297-specific peptides. *P* values were derived from Dunn's multiple comparison tests following Kruskal-Wallis tests.

c Peptides with index values >20.

10.1128/msphere.00252-22.1 TABLE S1 Serum IgM reactivity with DbpA peptide array. Download Table S1, DOCX file, 0.02 MB. Copyright © 2022 Movahed et al. 2022 Movahed et al. https://creativecommons.org/licenses/by/4.0/ This content is distributed under the terms of the Creative Commons Attribution 4.0 International license.

10.1128/msphere.00252-22.2 FIG S1 IgM and IgG reactivity with DbpA per patient sample (*n* = 263). The values (MFI) on the *y*-axis were determined by Luminex with DbpA-coated microspheres. Individual serum samples were probed using anti-IgM (left) or anti-IgG (right) secondary antibodies for each B. burgdorferi-seropositive cohort (as described in Materials and Methods). Download FIG S1, TIF file, 1.9 MB. Copyright © 2022 Movahed et al. 2022 Movahed et al. https://creativecommons.org/licenses/by/4.0/ This content is distributed under the terms of the Creative Commons Attribution 4.0 International license.

### Reactivity of B. burgdorferi-seropositive serum samples with DbpA peptide array.

To identify linear B cell epitopes on DbpA of B. burgdorferi strains B31 (DbpA_B31_) and 297 (DbpA_297_), we generated 18-mer peptide libraries that encompassed each of the DbpA variants. The DbpA amino acid sequences from B. burgdorferi strains B31 and 297 are 89% identical (16). As such, the final library consisted of a total of 31 peptides: 8 shared between strains B31 and 297, 12 specific to DbpA_B31_, and 11 specific to DbpA_297_ (Fig. 2).

**FIG 2:**
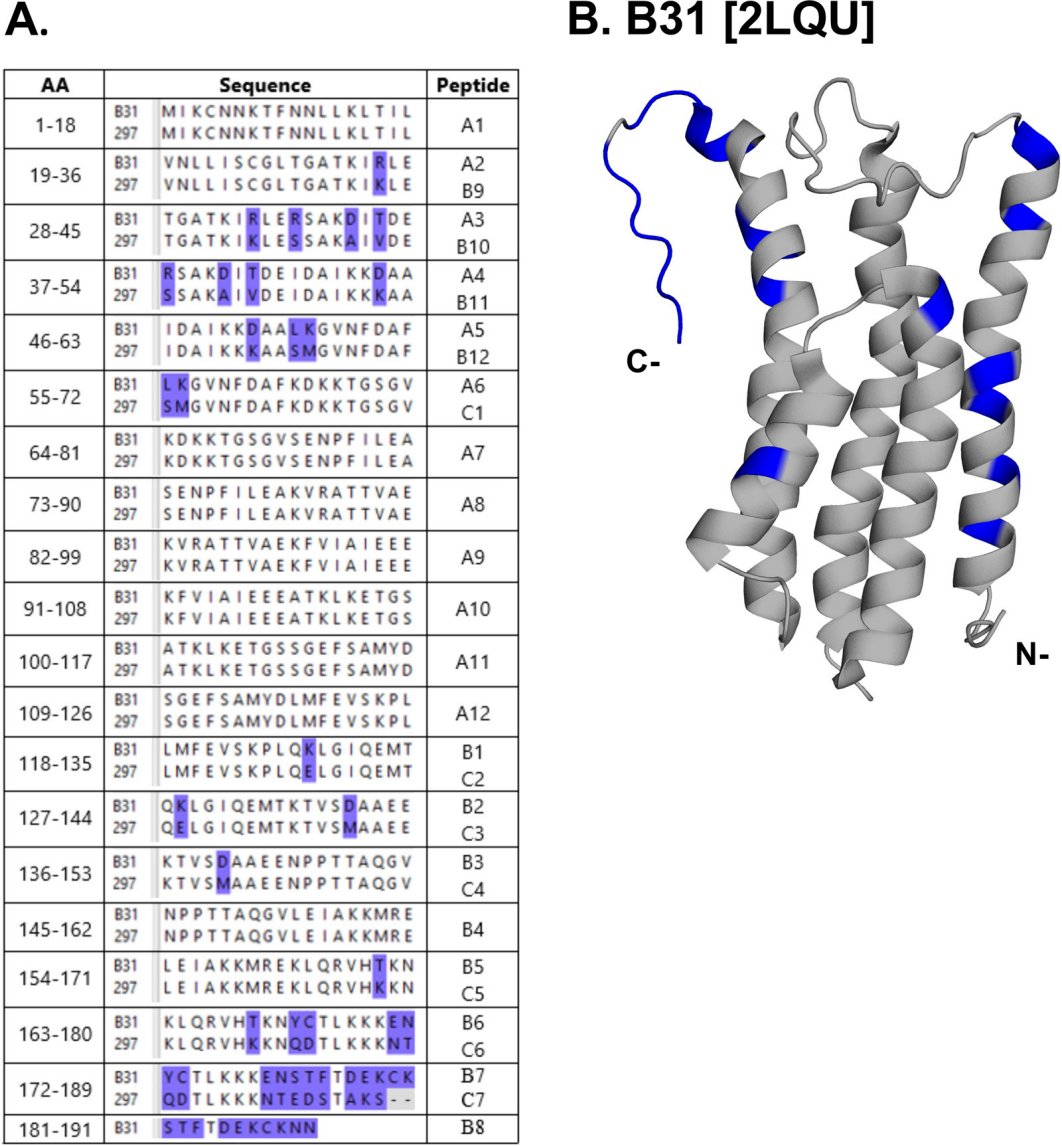
DbpA peptide arrays for B. burgdorferi strains B31 and 297. (A) Tabular alignment of DbpA amino acid number (left column), sequences (middle column), and corresponding peptide names for B. burgdorferi strains B31 (OspC Type A) and 297 (OspC Type K), as described in Materials and Methods. Residue differences between B31 and 297 are highlighted in purple in the table and are (B) illustrated using PyMol and PDB ID 2LQU on the structure of DbpA_B31_. The final array contained 31 peptides: 8 that were identical between sequences, 12 that represented B31 sequences, and 11 that represented 297 sequences.

To assess which (if any) DbpA-derived peptides are reactive with B. burgdorferi-seropositive sera, the peptides were coated onto 96-well microtiter plates and probed with control (*n* = 4) or seropositive (*n* = 23) human serum samples. IgM reactivity in the serum samples was limited to a few peptides, while IgG reactivity was much more pronounced, with ~60% of the peptides displaying above background reactivity (Fig. S2). Based on this cumulative reactivity profile by ELISA, a dozen DbpA peptides were chosen for detailed analysis by Luminex with our larger B. burgdorferi-seropositive serum sample set. Included in our down-selection were peptides identified by the Bepipred linear epitope prediction tool as having a high likelihood of being a target of antibodies (e.g., residues spanning 55 to 78, 101 to 113, 126 to 136, and 165 to 187).

10.1128/msphere.00252-22.3 FIG S2 Serum IgG pepscan analysis of DbpA. Microtiter plates were coated with the indicated peptides, then probed with B. burgdorferi-seropositive serum samples (*n* = 23) for IgG reactivity. The plates were developed with TMB (as described in Materials and Methods), and the cumulative absorbance for each peptide is plotted. Background reactivity, defined by antibody reactivity to block alone, was subtracted from each well. The cumulative OD_450_ values were then plotted on a 3D Area Chart (Microsoft Excel) and arranged from left to right by DbpA residue numbers. DbpA_B31_ peptides are displayed in blue, and DbpA_297_ peptides are displayed in orange. Download FIG S2, TIF file, 1.9 MB. Copyright © 2022 Movahed et al. 2022 Movahed et al. https://creativecommons.org/licenses/by/4.0/ This content is distributed under the terms of the Creative Commons Attribution 4.0 International license.

However, before performing the Luminex analysis, we examined antibody reactivity by ELISA with peptide A1 (DbpA residues 1 to 18) in more detail, as a peptide spanning residues 6 to 30 was recognized by sera from patients clinically diagnosed as having erythema migrans (EM), an early manifestation of Lyme disease (28). In our study, IgM and IgG reactivity with peptide A1 was 2- to 4-fold elevated over background, but this was only observed in a fraction (14 to 16%) of the samples tested (Fig. S3). Thus, our results are similar to those of Arnaboldi and colleagues in terms of IgM reactivity with peptide A1, but they differ in that we observed IgG reactivity with the same peptide. This discrepancy in IgG reactivity (low in the previous study, and detectable in our study) may simply be reflective of different patient populations, as one represented early-stage disease and the other a (two-tier positive) later stage. Overall, however, A1 antibody reactivity was considered low and was not included in the Luminex analysis.

10.1128/msphere.00252-22.4 FIG S3 IgM and IgG reactivity with A1 peptide in B. burgdorferi-seropositive serum samples. The optical density (OD) of A1 reactivity with IgM and IgG antibodies in 51 samples (A) in total and (B) by individual samples. We evaluated a total of 51 B. burgdorferi-seropositive serum samples for IgG and IgM reactivity and defined A1-positive samples as those which had ELISA values (OD) that were >2 SD above the average of three healthy controls. Download FIG S3, TIF file, 1.9 MB. Copyright © 2022 Movahed et al. 2022 Movahed et al. https://creativecommons.org/licenses/by/4.0/ This content is distributed under the terms of the Creative Commons Attribution 4.0 International license.

### Multiplex profiling of B. burgdorferi-seropositive serum reactivity with DbpA peptides.

Based on peptide array profiling by ELISA, 12 highly reactive peptides were synthesized with an N-terminal linker (-GGGSK) and a biotin-tag, then coupled to streptavidin-coated Luminex beads. We then performed a multiplex analysis on samples from all three clinical cohorts (IgM^+^/IgG^−^, IgM^+^/IgG^+^, and IgM^−^/IgG^+^). We established a healthy cutoff value (MFI) using a commercial panel of 87 serum samples that displayed low IgM and IgG MFI values for DbpA as well as the 12 DbpA peptides (Table 1; Fig. 1).

The Luminex analysis revealed that all 12 DbpA-derived peptides were recognized by IgG from at least one of the three B. burgdorferi-seropositive cohorts (IgM^+^/IgG^−^, IgM^+^/IgG^+^, and IgM^−^/IgG^+^). With two exceptions (peptides A7 and C7, which will be discussed below), the increase in peptide reactivities ranged from 0.4- to 15-fold over those of healthy controls (Table 1). Nine of the 12 peptides were also recognized by IgM from at least one of the three B. burgdorferi-seropositive cohorts (IgM^+^/IgG^−^, IgM^+^/IgG^+^, and IgM^−^/IgG^+^) (Table S1). Except for peptides A7 and C7, the IgM reactivities with the peptides were only marginally above background.

**FIG 3:**
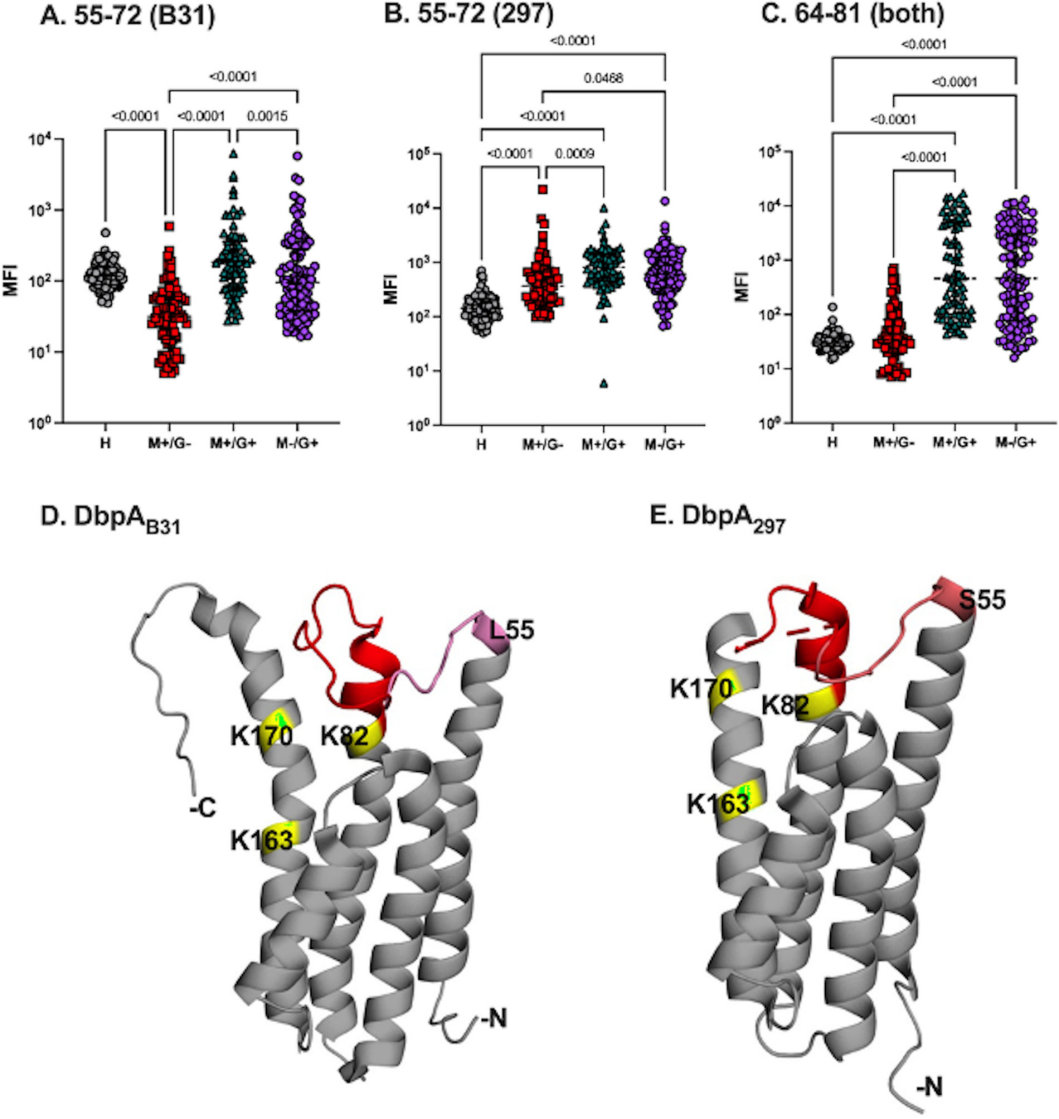
Reactivity of B. burgdorferi-seropositive serum samples with DbpA residues 55 to 81. IgG reactivity (MFI) in healthy (*n* = 86) or B. burgdorferi-seropositive serum samples defined as IgM^+^/IgG^−^ (*n* = 78), IgM^+^/IgG^+^ (*n* = 71), or IgM^−^/IgG^+^ (*n* = 114) for DbpA_B31_ and DbpA_297_ peptides spanning residues (A and B) 55 to 72 and (C) 64 to 81. Significance was determined by one-way ANOVA followed by Tukey’s post hoc test. (D and E) PyMol images of DbpA_B31_ (PDB ID 2LQU) and DbpA_297_ (PDB ID 4ONR) with residues 64 to 81 are colored firebrick red, and residues 55 to 63 are colored in shades of pink, with darker shades representing greater reactivity. The three lysine residues implicated in decorin binding (K82, K163, and K170) are colored yellow/green.

DbpA-derived peptides A7 and C7 stood out as being highly reactive with IgG in the B. burgdorferi-seropositive serum panel (Table 1) and moderately reactive within the IgM pool (Table S1). Specifically, IgG reactivity with peptide A7, which corresponds to DbpA residues 64 to 81, was not significantly elevated in the IgM^+^/IgG- serum panel, although it was 84-fold and 59-fold increased over background in the IgM^+^/IgG^+^ and IgM^−^/IgG^+^ sample sets, respectively (Table 1; Fig. 3). In the IgM fraction, A7 reactivity was significantly elevated (4- to 11-fold) in each of the three B. burgdorferi-seropositive cohorts (IgM^+^/IgG^−^, IgM^+^/IgG^+^, and IgM^−^/IgG^+^) (Table S1). The proximal peptide, corresponding to residues 55 to 72, was largely nonreactive for DbpA_B31_ (peptide A6) and moderately reactive for DbpA_297_ (peptide C1) (Table 1; Fig. 3). The reactivity of the distal flanking peptide (residues 73 to 90) was not examined by Luminex because the peptide (A8) was deemed nonreactive in our preliminary ELISA screen (Fig. S2) and was therefore not pursued further. Collectively, these results suggest that the A7 sequence constitutes an immunodominant epitope within itself.

**FIG 4:**
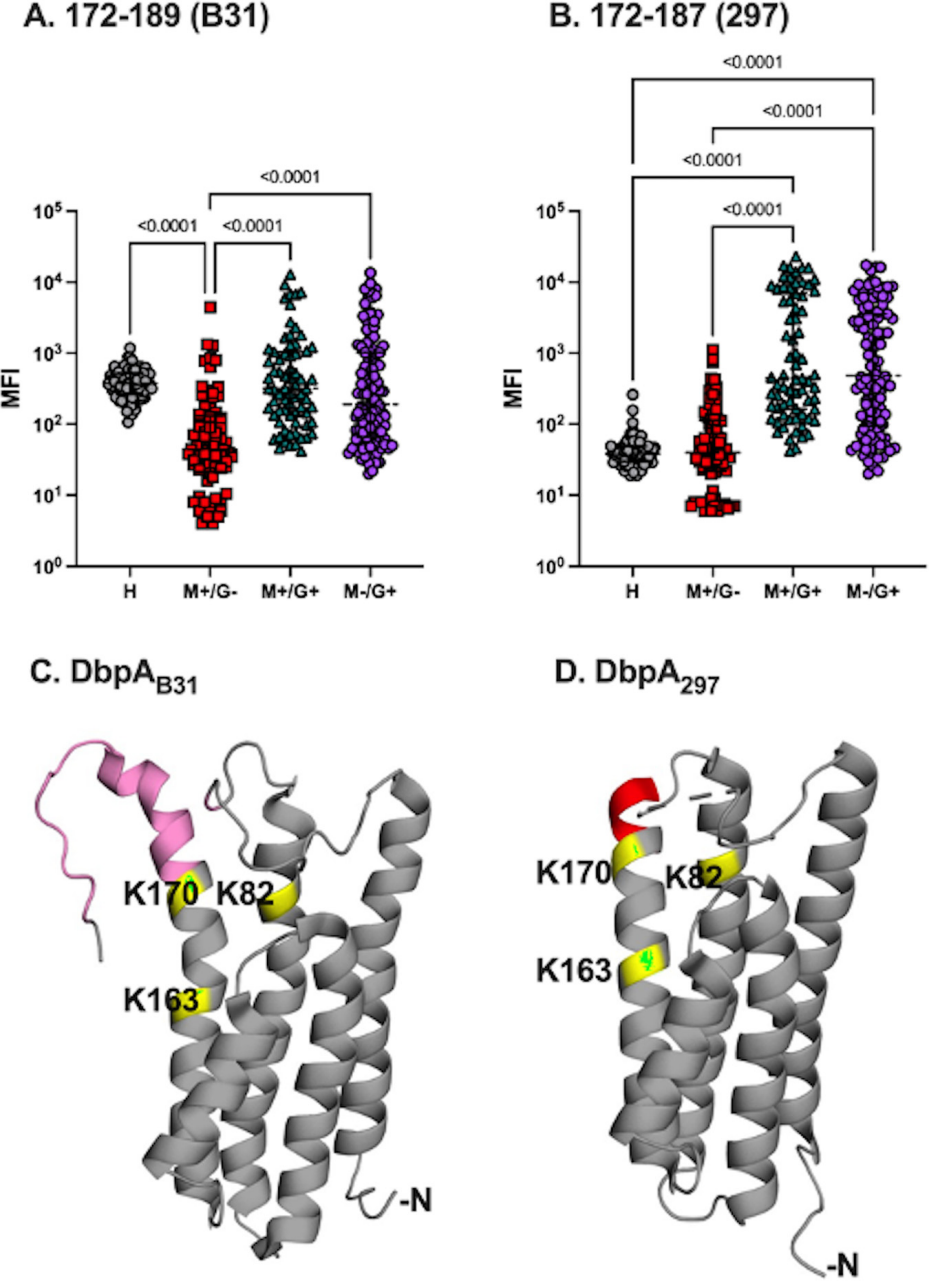
Reactivity of B. burgdorferi-seropositive serum samples with DbpA C-terminal residues. IgG reactivity (MFI) in healthy (*n* = 86), or B. burgdorferi-seropositive serum samples defined as IgM^+^/IgG^−^ (*n* = 78), IgM^+^/IgG^+^ (*n* = 71), IgM^−^/IgG^+^ (*n* = 114) for (A) DbpA_B31_ residues 172 to 189 and (B) DbpA_297_ residues 172 to 187. Significance was determined by one-way ANOVA followed by Tukey’s *post hoc* tests. PyMol images of (C) DbpA_B31_ (PDB ID 2LQU) with residues 172 to 189 colored pink and DbpA_297_ (PDB ID 4ONR) with residues 172 to 187 colored firebrick red. Residues 172 to 187 in DbpA_297_ are disordered and not visible in 4ONR. The three lysine residues implicated in decorin binding (K82, K163 and K170) are colored yellow/green.

The other notable peptide was C7, which corresponds to the extreme C-terminus of DbpA_297_ (residues 172 to 187). While C7 reactivity was not significantly elevated in the IgM^+^/IgG^−^ serum panel, it was 84-fold and 58-fold increased over background in the IgM^+^/IgG^+^ and IgM^−^/IgG^+^ sample sets, respectively (Table 1; Fig. 4). In the IgM fraction, C7 reactivity was significantly elevated (3- to 5-fold) in each of the three B. burgdorferi-seropositive cohorts (IgM^+^/IgG^−^, IgM^+^/IgG^+^, and IgM^−^/IgG^+^) (Table S1). These results clearly demonstrate that the 16 C-terminal residues of DbpA_297_ are targeted by antibodies in B. burgdorferi-seropositive samples.

However, we found it curious that the analogous C-terminal peptide (B7) from DbpA_B31_ (residues 172 to 189) was only weakly reactive by Luminex, compared to peptide C7 from DbpA_297_ (Fig. 4; Table S1), especially considering that B7 was one of the most reactive peptides in our preliminary ELISA screen (Fig. S2). Moreover, B7 is predicted to have a high propensity to be a linear epitope, according to Bepipred. We reasoned that the subdued response to B7 by Luminex might simply be related to the nature of the multiplexed bead array itself, for reasons related to interference or surface coupling (35). We therefore performed a comprehensive analysis of 30 control (healthy) and ~270 B. burgdorferi-seropositive samples for peptide B7 reactivity by ELISA. Antibody reactivity with peptide B7 was significantly higher in B. burgdorferi-seropositive serum samples, compared to that of healthy controls (*P* < 0.001) (Fig. S4), thereby confirming our earlier observation that the C-terminus (residues 172 to 189) of DbpA_B31_ constitutes an immunodominant linear epitope.

10.1128/msphere.00252-22.5 FIG S4 Healthy and B. burgdorferi-seropositive serum IgG reactivity with DbpA peptide B7. Microtiter plates were coated with B7 peptide (1 μg/well) in PBS (pH 7.4) and then probed with healthy (H) or B. burgdorferi-seropositive serum samples (1:100) before detection with horseradish peroxidase (HRP)-labeled goat anti-human IgG (as described in Materials and Methods). Statistical analysis was performed using a paired *t*-test (*P* < 0.0001) FIG S4, TIF file, 1.9 MB. Copyright © 2022 Movahed et al. 2022 Movahed et al. https://creativecommons.org/licenses/by/4.0/ This content is distributed under the terms of the Creative Commons Attribution 4.0 International license.

### Relationship between DbpA and peptide reactivity.

To determine whether reactivity to a particular peptide was simply proportional to overall DbpA antibody titers in any given individual, we determined the correlation coefficients between the DbpA MFI values and the peptide MFI values for each of the ~270 serum samples. As shown in Fig. S5, there was no notable correlation between the two variables, except for peptide A7, which had an R^2^ value approaching 0.4, indicating a weak to moderate relationship between DbpA and peptide-specific antibody levels. This observation raises the possibility that anti-peptide antibodies are elicited against DbpA breakdown products rather than intact (native) DbpA.

10.1128/msphere.00252-22.6 FIG S5 DbpA versus peptide reactivity in individual B. burgdorferi-seropositive serum samples. To examine the relationship between DbpA reactivity and individual DbpA peptides, we plotted the MFI from indicated peptide bead sets (on the *y*-axis) versus the MFI from DbpA (on the *x*-axis) for 293 samples, including healthy samples and those from the three B. burgdorferi-seropositive serum cohorts. Squared Pearson's correlation coefficients (*r*^2^) were calculated using GraphPad and are shown for each peptide. The best-fit lines for the regression are displayed as solid lines, while corresponding 95% confidence intervals are displayed as dotted lines. Download FIG S5, TIF file, 1.9 MB. Copyright © 2022 Movahed et al. 2022 Movahed et al. https://creativecommons.org/licenses/by/4.0/ This content is distributed under the terms of the Creative Commons Attribution 4.0 International license.

**FIG 5:**
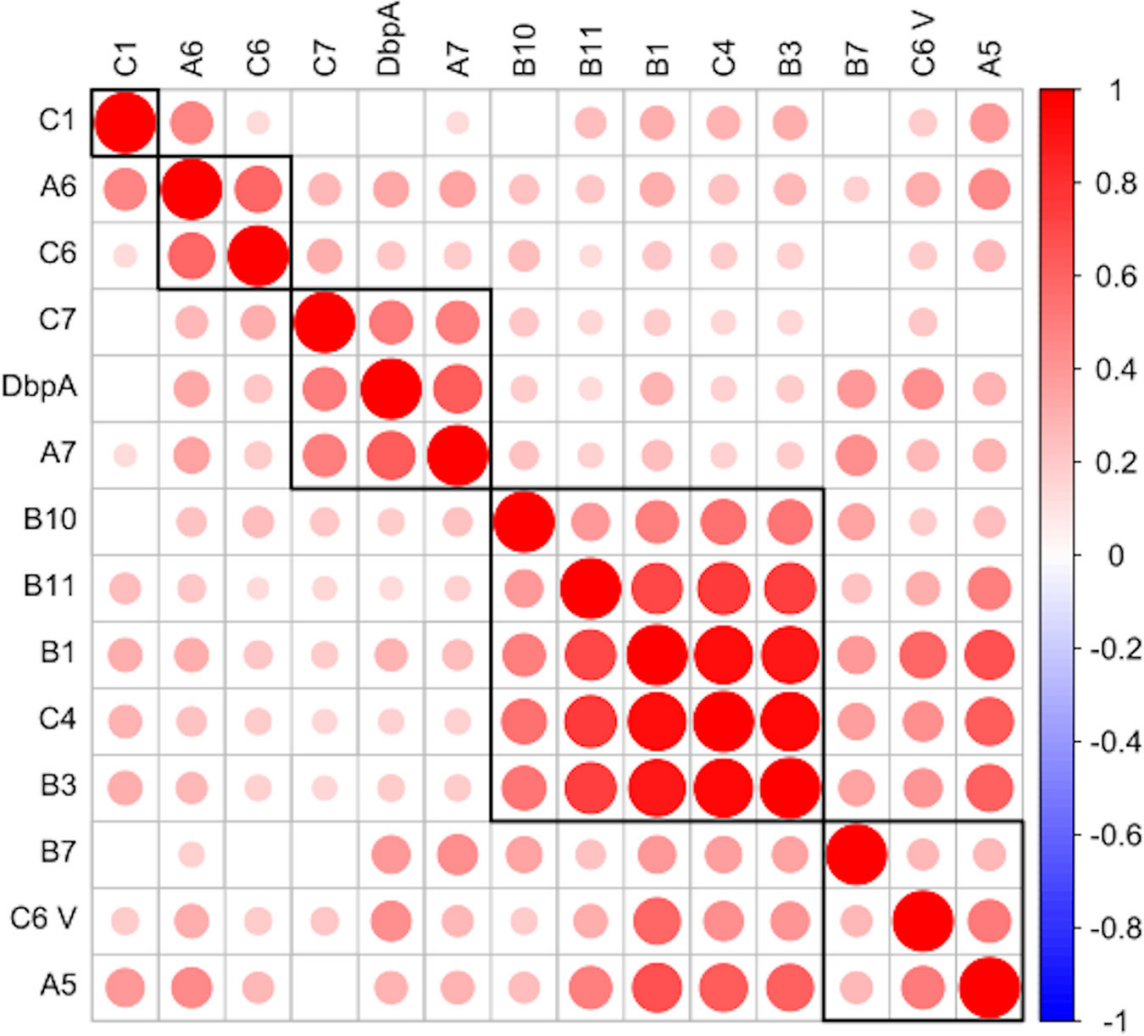
Correlation matrix of antibody responses to specific DbpA peptides. Pearson correlations were calculated for every possible combination of DbpA and peptide from the values presented in Table 1, and the resulting *P* values were adjusted for multiple comparisons by the Benjamini-Hochberg method. Correlations with significant adjusted *P* values are displayed in the matrices, while those with insignificant *P* values are left blank. The color (per scale on right) and relative size of each dot correspond to the strength of the corresponding correlation, and black rectangles around groups of correlations show the results of hierarchical clustering of the peptides.

To better assess the relationship between DbpA and peptide recognition, we examined correlations between available MFIs for all serum samples. A correlation matrix of serum IgG samples revealed four sets of peptides whose reactivity profiles tracked with each other, even though the peptides were not necessarily overlapping or even on adjacent regions of DbpA (Fig. 5). For example, the reactivities of A6 and C6 correlated with each other, as did those of C7, A7, and DbpA. The largest cluster consisted of B10, B11, B1, C4, and B3, which represent overlapping peptides (B10, B11), abutting peptides (B1, B3), and a solitary peptide (C4). The same correlations did not hold when IgM reactivity was examined, primarily because of much higher background values, which confounded our ability to sort out specific versus nonspecific relationships (data not shown). From this analysis, the correlation between the C7, A7, and DbpA reactivities was the most compelling, as those two peptides were identified as being highly reactive in the serum panel examined (Table 1).

### Native versus non-native linear B cell epitopes on DbpA.

**FIG 6:**
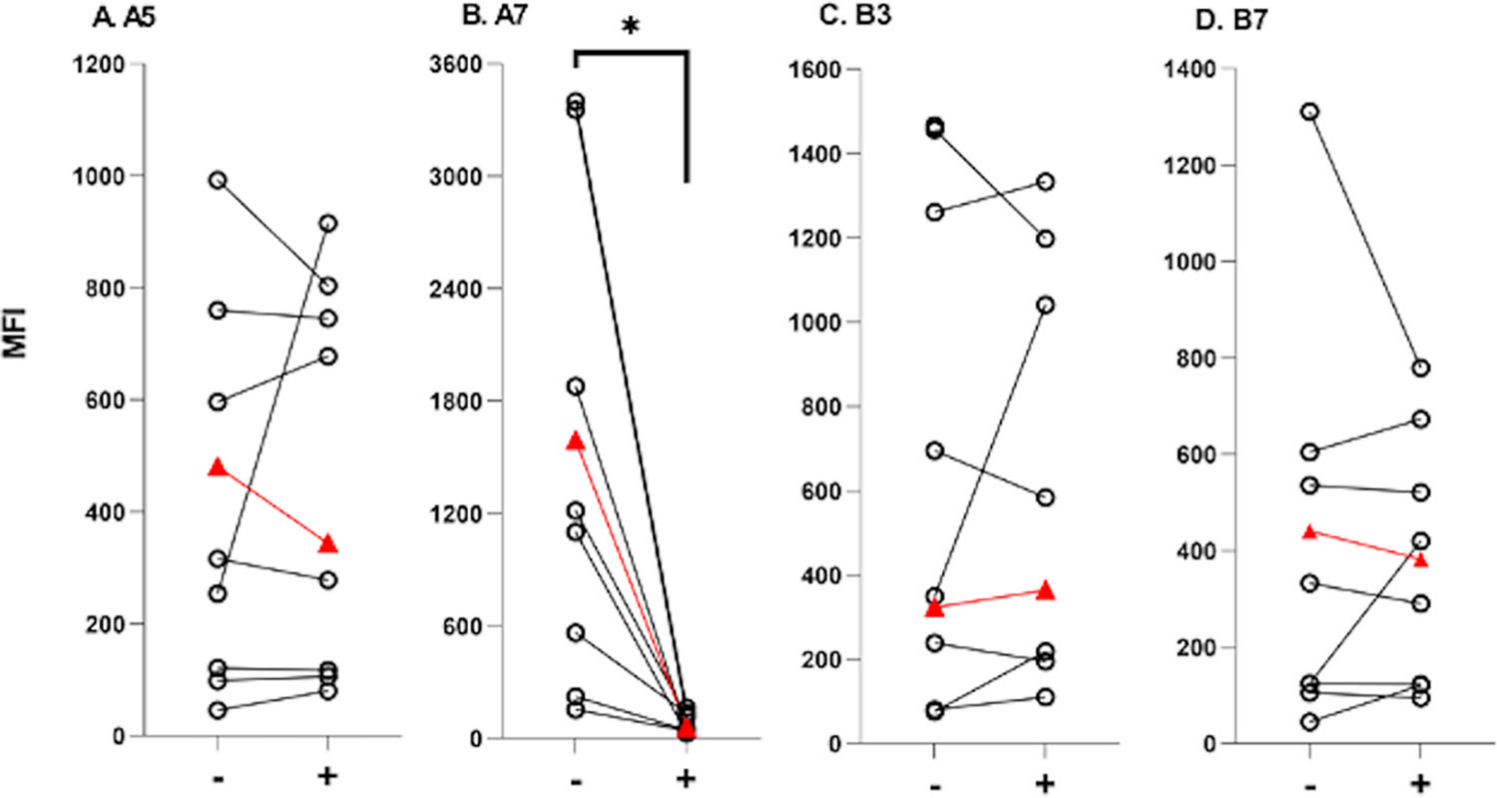
Recognition of recombinant DbpA by peptide-specific antibodies. B. burgdorferi-seropositive serum samples were incubated without (−) and with (+) soluble DbpA (10 μg/mL), then mixed with four different peptide-coated bead sets, as indicated in Panels A–D, and subjected to Luminex analysis. The relative reactivities (MFI; *y*-axis) without and with DbpA are plotted with individual samples connected by a line. Included in the analysis is an Accurun (+) sample indicated by the triangle (red). Only the reactivity of peptide A7 was reduced by the addition of soluble DbpA, as reflected by a reduction in MFI in the (+) column compared to the (−) column. Significance was determined by paired, two-tailed *t*-tests with Welch’s corrections. *, *P* ≤ 0.05.

The elicitation of antibodies against linear epitopes on a given protein antigen can occur in the context of the antigen’s native conformation (e.g., by being displayed on the surface of the pathogen) or non-native conformation, induced upon antigen release and/or degradation from the pathogen (36). In an effort to distinguish between these two categories in the case of DbpA, we reprobed four different peptide-coated microspheres in the absence and presence of a soluble recombinant DbpA (10 μg/mL) competitor. We reasoned that the reactivity of human antisera with a native linear epitope would compete with soluble recombinant DbpA, whereas reactivity to cryptic or non-native linear epitopes would not. Analysis of a subset of human serum samples revealed an immediate trend. Specifically, the addition of soluble DbpA had little or no inhibitory effect on antisera reactivity with A5, B3, and B7-coated beads (Fig. 6). Peptides B11 and C7 served as controls for these studies, as competition was not expected from them in the first place, since these peptides are derived from the B. burgdorferi strain 297 sequence and are sufficiently divergent from the DbpA_B31_ sequence (data not shown). Peptide A7 was different in that antibody reactivity was uniformly eliminated upon the addition of soluble DbpA in the B. burgdorferi-seropositive serum samples tested. We interpret these results as an indication that peptide A7 (residues 64 to 81) constitutes a surface exposed linear B cell epitope on DbpA, while epitopes A5 and B3 are cryptic in nature, with the caveat that we cannot fully exclude the possibility that the recombinant DbpA used for the competition studies does not necessarily reflect the native antigen when displayed on the spirochete surface.

## DISCUSSION

DbpA is one of the most antigenic outer surface proteins of B. burgdorferi, and, as such, has important implications for LD diagnostics and immunity (13, 14, 34, 37, 38). Indeed, antibodies against DbpA have been shown to promote the resolution of early B. burgdorferi infections (11). However, despite DbpA’s importance as an immune target, little is known about the specific epitopes on DbpA that are recognized by humans. To begin to address this question, we profiled a collection of ~270 archived human B. burgdorferi-seropositive serum samples for reactivity with a DbpA peptide array derived from two geographically representative B. burgdorferi type strains, B31 (OspC Type A) and 297 (OspC Type K). Twelve of the original 31 peptides reactive by ELISA were resynthesized with biotin-tags to enable multiplex analysis against the full suite of healthy and B. burgdorferi-seropositive sera (IgM^+^/IgG^−^, IgM^+^/IgG^+^, and IgM^−^/IgG^+^) in our collection. While the results revealed significant IgG (and some IgM) reactivity against essentially all of the peptides examined, two stretches of DbpA stood out as being highly immunoreactive, defined as displaying a >20-fold increase over the reactivity observed in healthy controls. The first stretch corresponds to DbpA residues 64 to 81 (peptide A7), an amino acid sequence conserved between DbpA_B31_ and DbpA_297_. The second corresponds to the respective C-terminal peptides of DbpA_B31_ (B7; residues 172 to 189) and DbpA_297_ (C7; residues 172 to 187), which are notably divergent from each other. Identification of these linear epitopes on DbpA may have utility in both Lyme disease diagnostics and, potentially, a next-generation Lyme disease vaccine.

DbpA residues 64 to 81 (peptide A7) are conserved between B. burgdorferi strains B31 and 297. When mapped onto the solution structure of DbpA_B31_ (PDB ID 2LQU), as shown in Fig. 3, the A7 peptide corresponds to the flexible loop between DbpA’s α-helix 1 and α-helix 2 in proximity to three key lysine residues (K82, K163, and K170) associated with heparin and decorin binding (16, 19). The peptide is similarly positioned on DbpA_297_, although the actual loop is disordered in the DbpA_297_ crystal structure (PDB ID 4ONR). In the case of DbpA_B31_, the two flanking peptides (A6 and A8) were only mildly reactive in our arrays, as was the flanking C1 peptide (residues 55 to 72) for DbpA_297_, suggesting that the central loop itself, rather than the adjacent α-helices, is the most immunoreactive. Interestingly, Tokarz and colleagues identified this same region as being reactive with antisera from patients with Lyme neuroborreliosis (IEDB ID 745110) (29). Thus, we propose that the flexible loop between α-helix 1 and α-helix 2 (and certain flanking residues) constitutes an immunodominant linear epitope on DbpA that is recognized in B. burgdorferi-seropositive patients and possibly in LD. Indeed, the same loop and flanking regions are predicted to contain conformational B cell epitopes, according to Discotope (39) and ElliPro (40). Finally, it is also noteworthy that the A7 peptide contains a tripartite motif (Thr-Gly-Ser) that is conserved in DbpA and DbpB from B. burgdorferi and B. garinii, although the functional significance of this motif is unknown (41).

The flexible linker between α-helix 1 and α-helix 2 has been implicated in influencing DbpA’s affinity for GAGs, raising the possibility that antibodies directed against the A7 peptide epitope block the interaction of DbpA and substrate (42–44). In the case of DbpA from B. burgdorferi strain N40, shortening the linker via the deletion of residues 62 to 71 resulted in DbpA having a ~2-fold increased affinity for heparin and dematan sulfate, an observation that is consistent with the loop physically occluding the GAG binding pocket (43). Hook and colleagues have argued that DbpA_B31_ and DbpA_297_ residues 76 to 90 (corresponding to peptides A7 and A8 in our array) contain a decorin binding site of their own. Specifically, they reported that a soluble peptide encompassing residues 76 to 90 (PFILEAKVRATTVAE) was sufficient to competitively inhibit biotin-labeled DbpA from adhering to immobilized decorin (45). In addition, antiserum raised against this peptide reduced DbpA-decorin binding by ~50%. Collectively, these results argue that human antibodies directed against peptide A7 would partially, if not completely, inhibit DbpA attachment to the ECM. Those same A7-specific antibodies would also presumably promote complement-mediated borreliacidal activity (6, 18, 27, 46). We are actively pursuing these hypotheses and have already demonstrated that the immunization of mice with an A7-KLH conjugate gives rise to DbpA-specific antibodies, confirming that residues 64-87 constitute a native linear epitope on DbpA. (E. Movahed and N. Mantis, unpublished results).

Peptides B7 (residues 172 to 189) and C7 (residues 172 to 187) from DbpA_B31_ and DbpA_297_, respectively, are also potentially consequential antibody targets, considering that the C-terminus of DbpA has been proposed to contribute to attachment to GAGs (15). Specifically, the C-terminal residues of DbpA are lysine-rich, with DbpA_B31_ and DbpA_297_ sharing a common KKK core motif (residues 176 to 178). Benoit and colleagues demonstrated that DbpA truncations were properly displayed on the spirochete outer surface but that resulting strains were unable to attach to 293 cells or immobilized GAGs (15). The available structures suggest that the DbpA’s C-terminus projects away from the bacterial surface and is readily accessible to substrates (and antibodies). In our study, peptide C7 was highly reactive with B. burgdorferi-seropositive serum, as measured by Luminex and ELISA, whereas the B7 peptide was reactive by ELISA but not Luminex. It is unclear whether the difference in B7 reactivity is due to the surface charge associated with polystyrene beads versus polystyrene Maxisorb ELISA plates or whether the biotin-tagged B7 peptide used for Luminex assumes a conformation not conducive to antibody recognition. Sorting this issue out has obvious implications, should B7 and other similar peptides be used as possible diagnostic markers for serology (37, 47).

In conclusion, this study represents an early effort to begin to better understand the human antibody response to DbpA and the role anti-DbpA antibodies play in resolving infection (48, 49). Certainly, the immunogenic nature of DbpA alone is of interest in terms of defining which pathogen-associated proteins stimulate B cell activation in humans and why this occurs (48, 49). DbpA is similarly immunoreactive in experimentally-infected Rhesus macaques and other species (12). In mice, for example, DbpA has been shown to be a T cell-independent antigen, indicating that it has the capacity to activate B cells directly in the absence of CD4 T helper cells (26). Whether this also applies to humans and/or contributes to the antigenicity of DbpA remains an open question. It is also unclear to what degree antibodies are elicited to native DbpA, as displayed on the bacterial surface, or to conformations of DbpA that may arise following bacterial lysis and protein release (50).

## MATERIALS AND METHODS

### Chemicals and biological reagents.

Chemicals and reagents were obtained from Thermo Fisher, Inc. (Waltham, MA), unless noted otherwise. PBS was prepared by the Wadsworth Center’s Cell and Tissue culture core facility.

### Cloning, expression, and purification of recombinant DbpA.

DbpA from B. burgdorferi B31 (NCBI: txid224326) was expressed in E. coli BL21 (DE3). The PCR amplicons for DbpA residues 26 to 188 were subcloned into the pNYCOMPS-C-term expression vector, encoding a C-terminal deca-His tag. The transformed E. coli BL21 (DE3) strain was grown at 37°C in TB medium until mid-log-phase (0.6 at OD_600_), after which it was treated with 0.1 mM IPTG and cultured for 16 h at 20°C. The cells were harvested by centrifugation and resuspended in 20 mM Tris-Cl (pH 7.5) and 150 mM NaCl. The cell suspension was sonicated and centrifuged at 30,000 × *g* for 30 min. After centrifugation, the protein-containing supernatant was purified by nickel-affinity and size exclusion chromatography on an AKTAxpress system (GE Healthcare), which consisted of a 1 mL nickel affinity column followed by a Superdex 200 16/60 gel filtration column. The elution buffer consisted of 0.5 M imidazole in binding buffer, and the gel filtration buffer consisted of 20 mM HEPES pH 7.6, 150 mM NaCl, and 20 mM imidazole. Fractions containing pure DbpA were pooled and subjected to TEV protease cleavage (1:10 weight ratio) for 3 h at room temperature to remove the deca-His tag. The cleaved protein was passed over a 1 mL Ni-NTA agarose (Qiagen) gravity column to remove the TEV protease, deca-histidine tag, and any uncleaved protein. DpbA was then buffer exchanged into 20 mM HEPES (pH 7.5) and 150 mM NaCl.

### Prediction of DbpA linear B cell epitopes.

The sequence of DbpA from B. burgdorferi strains B31 (DbpA_B31_; UniProt ID O50917) and 297 (DbpA_297_; UniProt ID Q1W5I8) were analyzed using the linear B cell epitope prediction tool Bepipred (set threshold 0.5) (30), available via the Immune Epitope Database (IEDB.org) (31).

### DbpA peptide array.

The DbpA sequences from B. burgdorferi strains B31 (OspC Type A; NCBI: txid224326) and 297 (OspC Type K; NCBI: txid521009) are ~89% identical. Peptide arrays covering DbpA from B. burgdorferi strains B31 and 297 were designed based on NCBI taxonomy ID sequences, as noted above, and synthesized by NeoScientific (Woburn, MA). The final library consisted of 31 peptides, of which 8 were identical between sequences, 12 were specific to B31, and 11 were specific to 297 (presented in Fig. 2). Each peptide was 18 amino acids in length and overlapped with the previous peptide by 9 residues, with the omission of a single peptide corresponding to residues 10 to 27, which failed Q/C. The peptides were solubilized in dimethyl sulfoxide (DMSO) at 10 mg/mL, and aliquots were stored at −20°C. Aliquots were thawed as needed and diluted in PBS (1 to 10 μg/mL) for routine use. Of the 31 original peptides, 12 that were reactive with a subset of B. burgdorferi-seropositive samples were ordered with a C-terminal GGGSK extension that was biotinylated on the terminal lysine (Genemed Synthesis, San Francisco, CA).

### Commercial and B. burgdorferi-seropositive serum samples.

Commercial Lyme disease seronegative (Lot 10500586) and seropositive (Lot 10510438) pooled samples were used as controls throughout this study (ACCURUN 810 and 130, respectively; SeraCare, Milford, MA). Healthy controls consisted of a commercial panel of 87 serum samples collected in 2017 and 2018 (Access Biologicals, Vista, CA). Primary clinical samples were obtained from the Wadsworth Center’s Diagnostic Immunology Laboratory. Those samples were submitted for Lyme disease serology and subjected to two-tiered testing consisting of (Tier 1) a C6 peptide screen (Immunetics; C6 Lyme ELISA) or Enzyme Linked Fluorescent Assay (ELFA; bioMérieux, VIDAS Lyme IgG II, and Lyme IgM II; Durham, NC) followed by (Tier 2) IgM and IgG detection by Western blotting (MarDX; Trinity Biotech, Carlsbad, CA). B. burgdorferi-specific IgM reactivity was defined as ≥2 positive bands, with IgG reactivity defined as ≥5 positive bands. Serum samples were aliquoted, de-identified, and classified as IgM-positive/IgG-negative, IgM-positive/IgG-positive, or IgM-negative/IgG-positive, based on the Western blot results. For this study, we employed a total of ~270 serum samples.

### ELISA and preliminary pepscan analysis.

Nunc Maxisorb F96 microtiter plates (Thermo Fisher Scientific) were coated with DbpA (0.1 μg/well) or DbpA peptides (1.0 μg/well) in PBS (pH 7.4), then incubated overnight at 4°C. The plates were washed three times with PBS-Tween 20 (PBS-T; 0.1%, vol/vol) and blocked with goat serum (2%, vol/vol, in PBS-T) for 2 h at room temperature before being probed with serum samples (1:100 dilution). Plate bound antibodies were detected with horseradish peroxidase (HRP)-labeled goat anti-human IgG or IgM polyclonal antibodies (SouthernBiotech, Birmingham, AL). The plates were developed with 3,3, 5,5-tetramethylbenzidine (TMB; Kirkegaard & Perry Labs, Gaithersburg, MD) and analyzed using a SpectroMax 250 spectrophotometer (Molecular Devices, Sunnyvale, CA).

### Multiplexed DbpA and peptide microsphere immunoassays (MIA).

Recombinant DbpA (5 μg) was coupled to Magplex-C microspheres (1 × 10^6^) using sulfo-NHS (N-hydroxysulfosuccinimide) and EDC (1-ethyl-3-[3-dimethylaminopropyl] carbodiimide hydrochloride), as recommended by the manufacturer (Luminex Corp., Austin, TX). Coupled beads were diluted in storage buffer (phosphate-buffered saline [PBS] with 1% bovine serum albumin [BSA], 0.02% Tween 20, 0.05% azide, pH 7.4) to a concentration of 1 × 10^6^ beads/mL.

Biotin-labeled peptides were complexed to Megaplex-avidin microspheres, following protocols provided by the manufacturer (Luminex Corp.). Briefly, microspheres were washed and resuspended in 250 μL of PBS-BSA, then subjected to vortexing and sonication. A total of 1.0 × 10^6^ beads in PBS-BSA were mixed with biotin-conjugated DbpA peptides (~5 μg) and incubated for 30 min at room temperature. The microsphere suspensions were then washed three times using a magnetic separator, resuspended in 500 μL of storage buffer, and stored at 4°C until use. Samples were analyzed using a FlexMap 3D instrument (Luminex Corp.).

### Statistical analysis.

ANOVAs and Student’s *t*-tests were carried out using GraphPad Prism, V 9.1 (Systat Software, San Jose, CA). Correlation matrices were prepared to examine correlations between MFI values of all responses to peptides, separated into IgG and IgM responses, using the R package corrplot (51).

### Molecular modeling.

The open-source molecular visualization software PyMol (DeLano Scientific LLC, Palo Alto, CA) was accessed at www.pymol.org and used for epitope modeling. Modeling was performed using DbpA structures PDB ID 4ONR (strain 297) and 2LQU (strain B31), available from the Protein Data Bank (rcsb.org) (19, 52).
