# Supplementary material for: Serological Analysis Identifies Consequential B Cell Epitopes on the Flexible Linker and C-Terminus of Decorin Binding Protein A (DbpA) from Borrelia burgdorferi
Source: mSphere. 2022 Jul 25;7(4):e00252-22. doi: 10.1128/msphere.00252-22 (PMC9429923; doi:10.1128/msphere.00252-22)
Supplement: TABLE S1 [file msphere.00252-22-st001.docx]

| Table S1. DbpA peptide reactivity by IgM from Lyme disease patient serum samples | | | | | | | | | | | |
| --- | --- | --- | --- | --- | --- | --- | --- | --- | --- | --- | --- |
|  |  | **Healthy** | **IgM+/IgG-** | | | **IgM+/IgG+** | | | **IgM-/IgG+** | | |
| AA*^a^* | **#***^b^* | **MFI (SD)** | **Index (SD)** | **MFI (SD)** | **p value** | **Index (SD)** | **MFI (SD)** | **p value** | **Index (SD)** | **MFI (SD)** | **p value** |
| 26-189 |  | 354.4 (286.1) | 6.8 (7.5) | 2429 (2661) | **<0.0001** | 4.9 (4.9) | 1757 (1770) | **<0.0001** | 4.4 (4.8) | 1563 (1729) | **<0.0001** |
|  |  |  |  |  |  |  |  |  |  |  |  |
| 18-45 | B10 | 1188 (1537) | 1.5 (1.4) | 1835 (1694) | **<0.0001** | 1.1 (1.1) | 1302 (1385) | 0.1861 | 0.7 (0.85) | 928.4 (1019) | >0.9999 |
|  |  |  |  |  |  |  |  |  |  |  |  |
| 37-54 | B11 | 884.8 (1307) | 2.1 (2.4) | 1860 (2177) | **<0.0001** | 1.6(1.8) | 1472 (1605) | **0.0001** | 1.2 (1.5) | 1128 (1356) | **0.0294** |
|  |  |  |  |  |  |  |  |  |  |  |  |
| 46-63 | A5 | 1593 (1764) | 1.4 (1.5) | 2297 (2425) | 0.0907 | 1.1 (1.3) | 1717 (2114) | >0.9999 | 0.51 (0.6) | 823.4 (985.3) | **<0.0001** |
|  |  |  |  |  |  |  |  |  |  |  |  |
| 55-72 | A6 | 947.2 (1049) | 1.7 (1.9) | 1660 (1813) | **0.0116** | 0.79 (1.0) | 752.6 (975.3) | 0.2087 | 0.50 (0.8) | 475.0 (763.8) | **<0.0001** |
|  | C1 | 1755 (1763) | 1.0 (1.1) | 1835 (2007) | >0.9999 | 0.8 (1.2) | 1529 (2205) | 0.0746 | 0.73 (0.8) | 1297 (1478) | **0.0035** |
|  |  |  |  |  |  |  |  |  |  |  |  |
| 64-81 | A7 | 172.8 (175.8) | 4.3 (7.1) | 748.2 (1227) | **<0.0001** | 11.4 (10.2) | 1975 (1769) | **<0.0001** | 9.1 (10.1) | 1576 (1753) | **<0.0001** |
|  |  |  |  |  |  |  |  |  |  |  |  |
| 118-135 | B1 | 1796 (1663) | 1.0 (1.2) | 1858 (2161) | >0.9999 | 0.3 (0.40) | 571.0 (733.5) | **<0.0001** | 0.21 (0.28) | 379.6 (513.0) | **<0.0001** |
|  |  |  |  |  |  |  |  |  |  |  |  |
| 136-153 | B3 |  |  |  |  |  |  |  |  |  |  |
|  | C4 | 1867 (2173) | 1.2 (1.2) | 2330 (2363) | 0.0859 | 0.88 (0.8) | 1653 (1589) | >0.9999 | 0.74 (0.82) | 1398 (1549) | 0.3471 |
|  |  | 1795 (1699) | 1.4 (1.3) | 2542 (2500) | 0.1405 | 1.1 (1.0) | 1993 (1939) | >0.9999 | 0.92 (1.0) | 1657 (1800) | 0.3024 |
| 163-180 | C6 |  |  |  |  |  |  |  |  |  |  |
|  |  | 72.07 (52.23) | 1.8 (1.6) | 129.4 (116.8) | **0.0211** | 0.8 (1.3) | 64.57 (95.33) | **0.0034** | 0.57 (0.55) | 41.35 (40.24) | **<0.0001** |
| 172-189 | B7 |  |  |  |  |  |  |  |  |  |  |
|  | C7 | 580.9 (746.0) | 3.589 (3.4) | 2085 (2019) | **<0.0001** | 1.856 (2.5) | 1078 (1455) | **0.0261** | 2.0(2.8) | 1181 (1655) | **0.0352** |
|  |  |  |  |  |  |  |  |  |  |  |  |
| *^a^*, amino acid residues; *^b^*,peptide number, as noted in Figure 2. Underlines indicate 297-specific peptides. Peptides with index values >45 are highlighted in red. | | | | | | | | | | | |
