# Supplementary figures and images for: Serological Analysis Identifies Consequential B Cell Epitopes on the Flexible Linker and C-Terminus of Decorin Binding Protein A (DbpA) from Borrelia burgdorferi
Source: mSphere. 2022 Jul 25;7(4):e00252-22. doi: 10.1128/msphere.00252-22 (PMC9429923; doi:10.1128/msphere.00252-22)

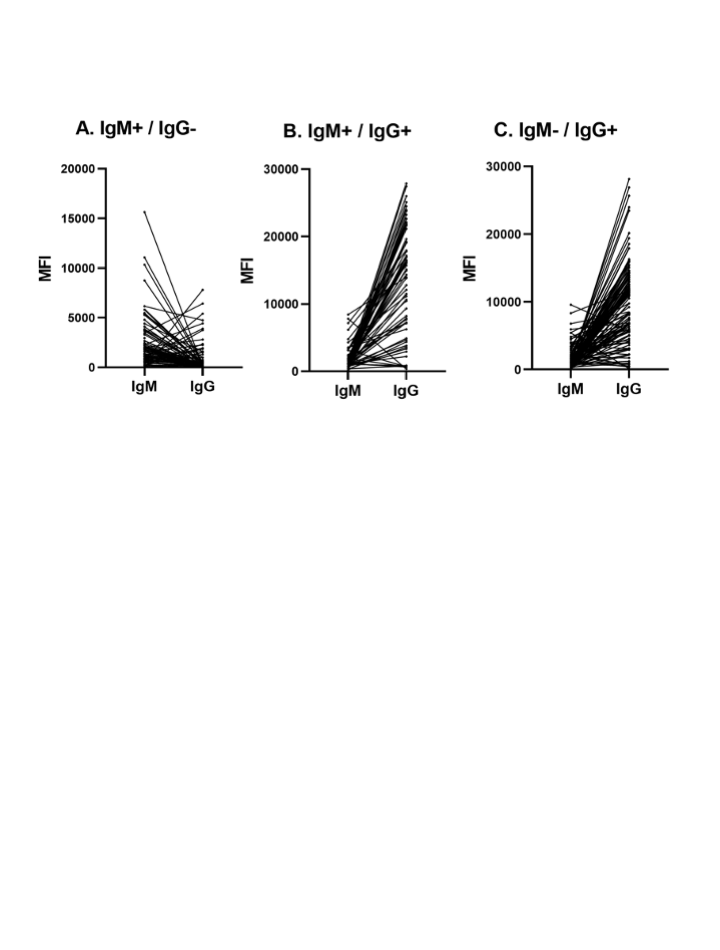

Supplement: FIG S1 [file msphere.00252-22-sf001.tif]

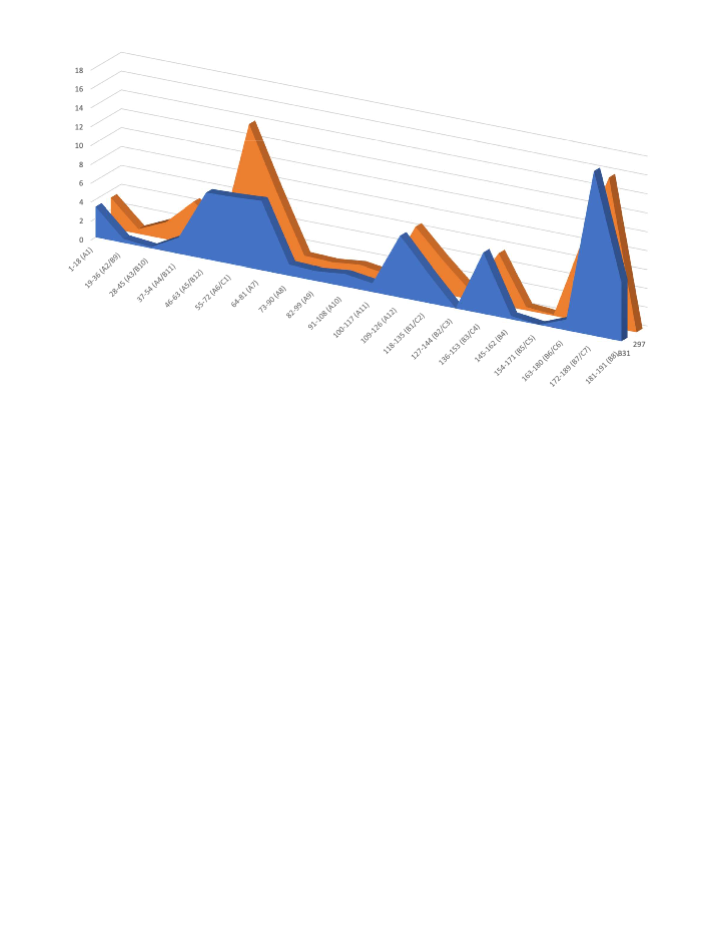

Supplement: FIG S2 [file msphere.00252-22-sf002.tif]

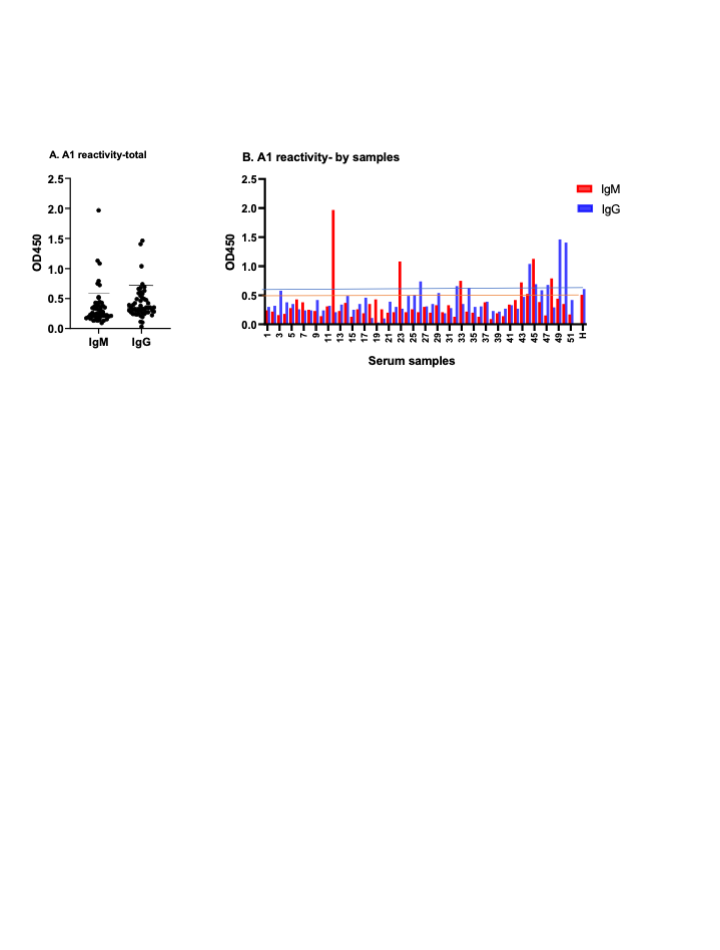

Supplement: FIG S3 [file msphere.00252-22-sf003.tif]

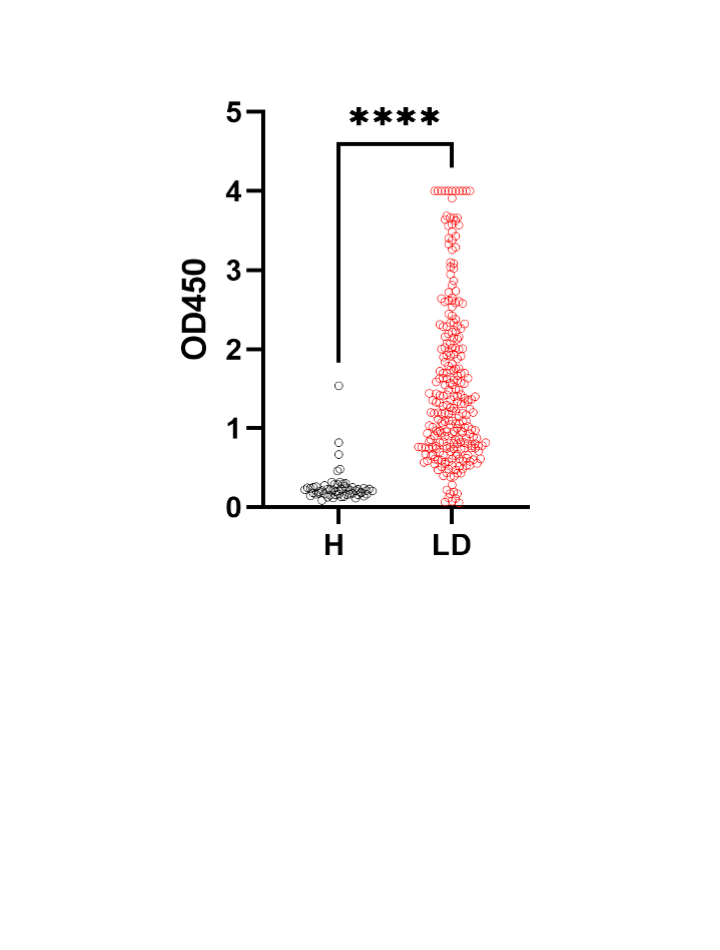

Supplement: FIG S4 [file msphere.00252-22-sf004.tif]

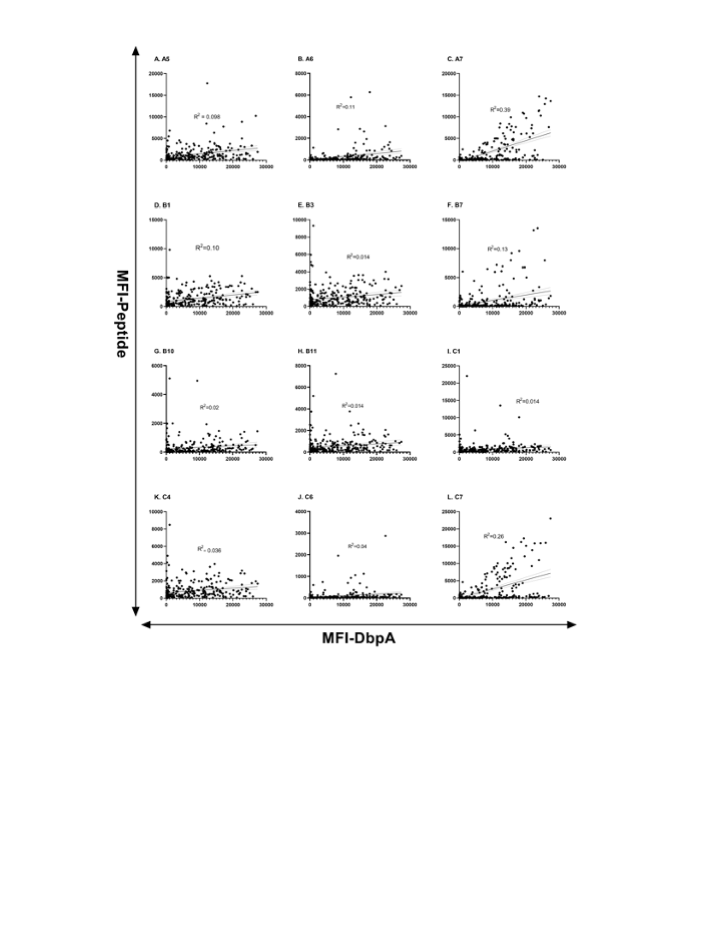

Supplement: FIG S5 [file msphere.00252-22-sf005.tif]
